# Influenza: Toward understanding the immune response in the young

**DOI:** 10.3389/fped.2022.953150

**Published:** 2022-08-19

**Authors:** Sonia Sakleshpur, Ashley L. Steed

**Affiliations:** Department of Pediatrics, Washington University School of Medicine, St. Louis, MO, United States

**Keywords:** influenza A virus, neonatal immune response, microbiota, immunity, antiviral immunity

## Abstract

Annually influenza causes a global epidemic resulting in 290,000 to 650,000 deaths and extracts a massive toll on healthcare and the economy. Infants and children are more susceptible to infection and have more severe symptoms than adults likely mitigated by differences in their innate and adaptive immune responses. While it is unclear the exact mechanisms with which the young combat influenza, it is increasingly understood that their immune responses differ from adults. Specifically, underproduction of IFN-γ and IL-12 by the innate immune system likely hampers viral clearance while upregulation of IL-6 may create excessive damaging inflammation. The infant's adaptive immune system preferentially utilizes the Th-2 response that has been tied to γδ T cells and their production of IL-17, which may be less advantageous than the adult Th-1 response for antiviral immunity. This differential immune response of the young is considered to serve as a unique evolutionary adaptation such that they preferentially respond to infection broadly rather than a pathogen-specific one generated by adults. This unique function of the young immune system is temporally, and possibly mechanistically, tied to the microbiota, as they both develop in coordination early in life. Additional research into the relationship between the developing microbiota and the immune system is needed to develop therapies effective at combating influenza in the youngest and most vulnerable of our population.

## Introduction

Influenza contributes significantly to morbidity and mortality globally and is especially devastating at the extremes of the population, the young and the elderly. Influenza causes an annual epidemic resulting in 3 to 5 million cases of severe illness and 290,000 to 650,000 respiratory deaths globally (1). Strikingly, about 99% of the deaths occur in children under the age of five in developing countries. Children under five who are otherwise healthy are at increased risk for influenza simply based on age. Children under age of two are additionally at higher risk for serious health complications due to influenza, such as pneumonia, encephalopathy, and the worsening of pre-existing chronic health problems. Moreover, among children under five, influenza-related outpatient hospital visits were 10 to 250 times as common as hospitalizations, highlighting the toll influenza takes on the young annually (2).

Influenza is not only dangerous for young children but the entire population as children are more likely to contract influenza and are more likely to transmit influenza to all age groups. Younger children tend to have longer periods of viral shedding over which they are contagious compared to adults (3).

Vaccination has been a key strategy to protect the population from influenza. Unfortunately, the young immune system responds to immunization with lower memory responses, resulting in lower antibody and memory T cells compared to adults (4). While unvaccinated infants are at an even greater risk, vaccinated infants have a reduced ability to prevent morbidity from influenza compared to other age groups (5).

Controlling and eliminating infant viral infections may also have far reaching health benefits and minimize the risk of childhood cancer as certain infections have been found to trigger the development of some cancers (6). While this link is well-established, it remains unclear if the differential immune response generated by the young contributes to this malignant cellular transformation. Therefore, further understanding of age dependent immune responses will likely inform across multiple fields of health care. Of note, in those patients already undergoing treatment for cancer, influenza poses a further threat given their immunocompromised status (7).

While recent studies investigating the influenza virus have begun to establish mechanisms behind the varied immune response in adults and the young, the infant's innate and adaptive immune system response to influenza is still poorly understood. While previously considered to be deficient, the young's differential response may serve a separate adaptive function. The infant's immune response may be tied to or instructed by the establishment of the microbiota. The microbiota has been shown to impacts the infant's immune function, and certain microbes are correlated with induction of specific cytokines and therefore play a role in resistance to infection (8). This relationship is worthy of further investigational exploration as it may lead to effective strategies to mitigate the impact of influenza in the young.

## Many viral infections have more severe pathogenesis in children than in adults

Viral infections in children are typically more severe and often have a different disease presentation than adults. These viral infections in children, specifically infants and toddlers, can impact the function of multiple organ systems and have fatal consequences. Their social behaviors make them more likely to contract viral infections in the first place. Children also have differences in anatomy that make airway clearance challenging during viral respiratory infections. Their smaller airways lend to mucous obstruction and their cartilaginous chest walls promote ease of collapse increasing the severity of pulmonary disease. Enteric viral illnesses that often cause asymptomatic or mild infections in adults can have life-threatening impacts on young children, specifically those under two, often via severe dehydration. While vaccines exist for many common viral illnesses, their effectiveness in infants and young children has been relatively limited (9).

The most prevalent viral infections in infants and children are respiratory viruses, and influenza is a leader in causing severe disease in young children. Studies have found children under the age of five have a 12-fold increased risk of hospital admission due to influenza virus infection compared to older children. While infants with predisposing medical conditions are at a greater risk for severe viral complications, healthy infants and young children have also been shown to develop severe cases of infection (10). Some studies have found that the number of influenza-related deaths of previously healthy children is almost equal to the number of deaths in children with chronic illnesses. Comparatively, most adults with fatal cases almost always had preexisting health conditions (11).

## Children's immune systems respond to infections differently than adult immune systems

Infants and young children are more susceptible to many viral infections and rely primarily on innate immunity, as their adaptive immune system remains naive due to limited pathogen exposure. The development and utilization of the innate immune system soon after birth aids in the development of the adaptive immune system. Studies have shown that neonatal innate responses may not be fully developed at birth, contributing to their increased susceptibility to infection (12). However, under some circumstances, neonates are able to mount a seemingly mature innate and adaptive immune response (13). The exact mechanisms underlying this differential response in the young are yet to be understood, but it is apparent that their innate and adaptive immune systems function differently from adults and is worthy of further investigation. This need is further highlighted by the COVID-19 pandemic, in which children have consistently fared better than their adult counterparts (14) and underscores the importance of understanding the cellular and molecular mechanisms driving these discrepant outcomes.

Interestingly, the differences in children's immune systems also vary geographically. Studies have shown ties between the innate immune system and the gut microbiome, which is heavily influenced by the geographic environment in which children are raised. The colonization of the gut by certain bacteria, partially determined by the predominant diet and lifestyle of the region, correlated with varied cytokine responses to the TLR2 pathway, production of IL-10 by dendritic cells, and the pro-inflammatory response (15–17).

## Age-related changes in innate immune response contribute to the increased morbidity in children with influenza

Our current understanding of virology does not readily predict differences in the mechanism by which influenza infects adults and children at the virus-cell level. Thus, the varied symptoms and differential pathogenesis in children and adults is likely the result of known differences in immune responses and functions.

Studies looking specifically at cytokine levels and innate immune function have found elevated levels of IL-6, IL-12, and IFN-γ in critically ill adolescents who survived influenza (18). Non-surviving adolescents had severely low levels of the specific inflammatory cytokine TNF-α, which is typically produced by innate immune cells in recovered adolescents. Congruently, infected children also had elevated levels of IL-6 (19). These findings have led some to believe that hypercytokinemia, the rapid release of many systemic inflammatory cytokines, during influenza infection may contribute to morbidity and mortality in the young (19). Specifically, children's predisposition to increased inflammation in response to influenza may be responsible for their severe disease progression which may contribute to influenza-related death observed worldwide (11).

Studies of innate immune responses in infants compared to adults found less responsive innate immune function. Activation of TLR expression in children and adults was equivalent, but the downstream effects of the TLR activation needed for viral clearance varied with age (20, 21). Infant monkeys exhibited increased viral replication and decreased type 1 IFN secretion (22). The importance of type I IFN is highlighted by recent studies demonstrating that the transcription factor RUNX1 facilitates influenza infection by dampening IFN responses (23) and further underscored by identification of mutations in the type I IFN pathway in patients with severe influenza (24, 25). In contrast to adolescents, the infant and child innate immune responses also had limited production of IL-12 and IFN-γ (26).

Neonatal mouse models of infection are able to recapitulate increased influenza-induced morbidity and similar immune responses (or lack thereof) as observed in human children. Mouse models have found that neonatal T cells in the lungs have lower IFN-γ secretion, a finding that extended past the neonatal period (27). Animal models have also shown that infected neonatal mice secrete less IFN-γ specifically from their NK cells, T cells, and macrophages (28, 29). Neonatal mice also exhibit delay viral clearance compared to adult mice (27). This delayed clearance is associated with low levels of CD4 T antiviral and helper cell activity due to blocks in cytokine production and IFN-γ signaling (27). The inefficient response of the young's immune system to influenza infections likely contributes to increased morbidity during their first few years of life (30).

Further studies looking at the differentially regulated genes in preterm and term children have shown similar trends. Specifically preterm children had comparatively higher expression of genes that downregulated IFN-γ production, IL-10 secretion, and T cell proliferation. These findings may explain the differences in susceptibility to influenza and other diseases between preterm and term infants (27, 31).

Conversely, as it pertains to SARS-CoV-2 infection, infants and children tend to exhibit milder symptoms and disease pathogenesis compared to adults (32). Infants lack the lung inflammatory response triggered by adult innate immune systems (33), possibly due to increased immunosuppression from a less reactive innate immune system (34, 35). The downregulated innate immune response in infants, that is a major factor in increased childhood influenza morbidity, may be protective during SARS-CoV-2 infection.

Taken together, both excessive damaging inflammation but deficient expression of certain mediators necessary to control viral replication may work in concert to negatively impact the young's response to many viral infections. Accordingly, recent studies aimed at identification of which aspects of the immune response contributed to increased illness severity found that the deficient innate immune response works in conjunction with a decreased adaptive immune response. Therefore, the young's adaptive immune system, specifically T-cell immunity, deserves further investigation and discussion.

## Neonatal adaptive immunity serves a differential not deficient function in responding to pathogens

Previous perceptions of the neonatal immune system as deficient are now being revised as studies are finding that the altered immune response to pathogens may serve a purposeful function. The lungs of infected neonatal mice have been observed to have lower levels of memory CD4+ and CD8+ T cells (36), which was initially viewed as a deficient function. This finding is cell-intrinsic, not environmentally regulated (5). The inability to generate memory cells may be an evolutionary advantageous adaptation, as it is more beneficial for survival to opt for a robust generalized response to infection rather than developing memory cells early in life (5).

Furthermore, CD8+ T cells made at birth have been found in adults with continued functionality (37). Neonatal adaptive immune cells may not respond to infections in the same manner as those of the adult immune systems because they serve a separate, developmentally important function which is maintained independently of adult immune function.

Mechanistically, neonatal T cells are placed in an effector-like state by developmentally regulated miRNAs, which have been found to modify and monitor their activation, differentiation, and metabolism (38–40). Adult immune cells, on the other hand, have greater potential to differentiate into memory cells and are less metabolically active (37, 41). Neonates express and display receptors typically associated with innate T cells and produce IL-8 that triggers a broad and non-specific response (42). This adaptation programs neonatal immune cells to respond to a wide range of pathogens, a response that is likely useful in the first few years of life.

## T regulatory cells help viral clearance independent of T cell migration patterns

Neonates have a high number of T regulatory cells (Tregs) in the lungs which monitor and modify immune responses. However, the lack of non-regulatory T cells moving into the alveoli of infected lungs occurs independently of Tregs, as neonatal mice deficient in Tregs do not have different T cell migration patterns (43).

Two regulatory cytokines, IL-10 and TGFβ, have been identified to play a direct role in mediating T cell migration into airways. IL-10 knockout pups with neutralization of TGFβ by antibody or with deletion of the TGFβ receptor have shown increased infiltration of CD4+ cells into the airways (43). However, there is conflicting evidence on the role of IL-10 in solidarity. Some studies have shown IL-10 knockout pups had no significant difference in T-cell migration or viral clearance while others found that IL-10 is necessary for the inflammatory response induced by influenza yet has a negative effect on viral clearance (43, 44). Whether these cytokines play a role in the differences in the young's response to infection is worthy of further investigation.

## Delayed migration of T cells into alveolar spaces in response to reduced expression of cytokines and preference for a type-2 response bias

While neonatal mice have difficulty recruiting T cells into the alveoli, they still have a delayed T cell response to influenza infection in the interstitial tissue. This delayed response corresponds with reduced signaling from proinflammatory cytokines TNF-α and IFN-γ (45). The alveolar spaces of neonatal lungs contain normal levels of neutrophils and elevated levels of eosinophils likely in response to elevated expression of chemokines CCL5, CCL3, and CXCL2, but lacks the T cells that were characteristic of infected adult lungs (46).

The bronchoalveolar lavage fluid of infected neonatal mice has reduced levels of IFN-γ and its induced chemokine CXCL9 (46). IFN-γ was not necessary to clear influenza from the lungs as IFN-γ knockout mice had no difference in influenza mortality, but there was an association between mice deficient in IFN-γ and the delayed T cell response (47). Additional studies that exogenously administered adult levels of IFN-γ to infected neonates were unable to elicit increased T cell migration into the alveolar spaces (46). Titration experiments conducted in adult mice found that T cell migration was independent of viral dose, and similar experiments in neonatal mice established that the lack of T cell migration into the lungs is independent of the viral load (46). Thus, IFN-γ may play a role in T-cell antigen presentation or priming that is necessary to clear infection.

T cell migration patterns may be influenced by the inability of neonatal mice to have a clear type-1 biased T cell response. Neonates seem to have a greater number of type-2 biased T cells that geographically cluster in areas without viral antigen, thereby contributing to the general interstitial inflammation observed in neonates (48). While specific chemokines may not be the singular defect in T-cell alveolar migration, there have been associations between the lack of certain chemokines and their cellular source in the young (49).

## γδ T cells initiate increased production of IL-17 as a part of a type-2 immune response which protects neonatal lungs from further damage

The decreased number of CD8+ T cells in the neonatal lungs in response to infection may be due to their inability to initiate a robust type-1 immune response. However, neonates have increased development and migration of γδ T cells to the lungs which may contribute to lung tissue homeostasis during infection and is associated with a type-2 immune response (50). A recent study found that certain γδ T cells can be triggered by viral infection to undergo transcriptional reprogramming in an adaptive manner like CD8+ T cells in response to antigen-induced differentiation (51). These changes in γδ T cells may be a beneficial immune response during viral infection.

γδ T cells produce IL-17A which initiates a type-2 tissue response with the production of IL-33, the recruitment of ILC2s and Treg cells, and the production of amphiregulin (52, 53). Specifically, IL-17A suppresses early IFN-γ expression in the lungs to establish a type-2 response. IL-17A, however, serves as a feedback mechanism by later negatively regulating type-2 cytokines to possibly prevent further tissue damage in the lungs (54, 55).

There is contradicting evidence on how IL-17A and influenza induces IL-33 production and the downstream effects triggered for tissue repair and remodeling. IL-33 is an alarmin cytokine that triggers type-2 inflammation, and mice lacking IL-33 have defective ILC2 responses and impaired immunity (56, 57). Children with influenza had elevated levels of IL-33, which positively correlated with levels of IL-17A but not with IFN-γ (52). IL-33 has not been shown to be associated with type-1 immune responses, albeit IL-17A may have an important regulatory role in fighting early neonatal infection. The cellular source of these key cytokines also impacts downstream biology as epithelia-derived IL-33 is pro-inflammatory while dendritic cell derived IL-33 is immunosuppressive (58). How IL-33 signaling and its outcome are regulated in the young vs. older population remains to be determined.

While the links between IL-17A and IL-33 pathways are unclear during influenza infection, they play an important role in type-2 immunity and mediating infection responses in the young. Given the smaller, more fragile lungs of neonates, lung damage from influenza can have severe short and long-term effects. The preference for a type-2 immune response over a type 1 immune response may be necessary to protect young lungs and prevent further damage (52). Therefore, the IL-17A/IL-33 pathway requires further study as its axis may be a potential target for therapeutic intervention.

## Neonatal immune system function and response are correlated with microbiota development

Further studies looking at the adaptation of infant immune systems to their environment have found connections between the gut microbiota and immune function. Similar to the immune system, the microbiota evolves with the host and adapts to its environment. Infant immune systems are influenced by early exposure to the microbiota after birth and are largely determined by the maternal microbiota. Early microbial colonization plays an important role in the immediate development of the immune system. Germ-free mice have abnormal immune development and deficient antibody production (59–61). Specifically, germ-free mice have lower numbers of IL-17, defective T regulatory cells, and impaired responses to certain inflammatory pathogens (59). A dysbiosis in the microbes colonizing human newborn gut has been shown to impede the development of a normal immune system early in life (31). Disturbances in microbiota development can also create long-term health impacts by resulting in immune defects. A lack of microbiota colonization can result in chronic inflammatory diseases and early administration of antibiotics in infants can lead to the development of diabetes and eczema (62).

While the exact mechanisms through which microbes impact immunity remains to be established, the microbiota clearly plays an important regulatory role in many aspects of immunity. Metabolites from the gut microbiota can impact the immune response directly on the mucosa or by entering circulation through epithelial cells (63). Gut microbes can produce fatty acids that impact the immune system or generate metabolites that bind to specific immune receptors (64). Pattern recognition receptors that play a role in innate immunity can respond to microbes and initiate cytokine release and signaling that further modulate immune responses (63).

The role of the developing microbiota in influenza-infected infants is still unclear. Given the correlation between neonatal immune development and microbiota colonization as well as the vast effects of the microbiota on immunity, further study is warranted to understand how the young's microbiota affects the differential response of the immune system to influenza. Such an understanding is particularly important as microbial interventions have the potential to confer beneficial immune responses and assist in targeted therapies (65).

## Conclusions

Infants are generally more susceptible to viral infections than adults and have more severe disease pathogenesis. Influenza in particular is a dangerous seasonal threat to the young population. However, their immune systems respond differently than adults, making existing interventions such as vaccines less effective. Deciphering differences in the young's immune response can therefore inform development of more targeted and effective therapies.

Given the known differences in their innate and adaptive immune responses, there is a growing consensus that neonate immune systems may serve a unique function and purpose compared to adults. Their more broadly generalized immune response and type 2 skewing may serve to protect the young in the first few years of life when multiple pathogens are likely to be encountered without also eliciting damaging inflammation. The well-observed differences in T cell migration patterns in infants is considered a major factor in their poor outcomes upon influenza infection. In addition, the differential expression of certain cytokines and stimulation of Tregs likely contributes as well.

**Figure 1:**
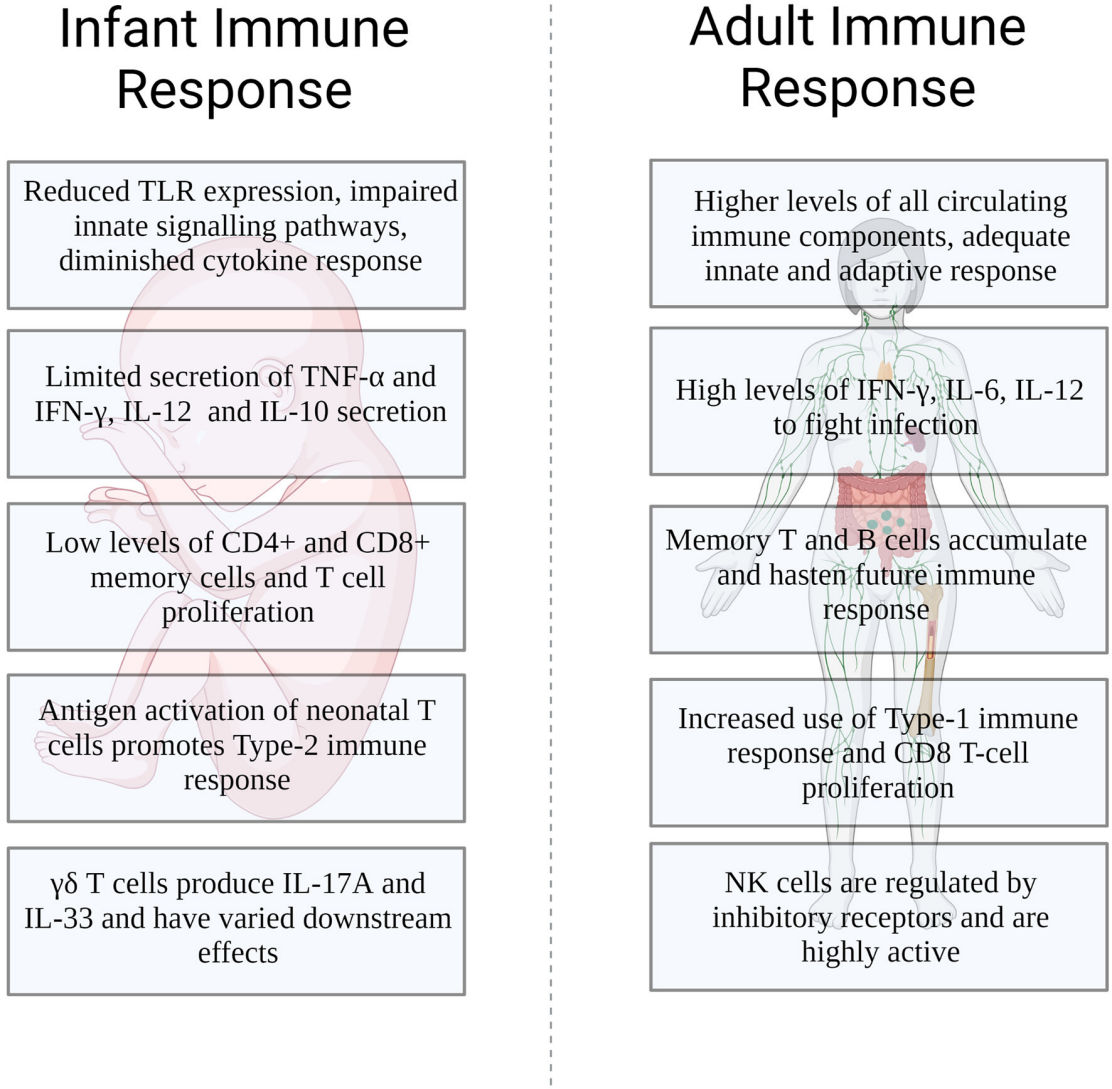
Differences in the infant and adult immune response to influenza infection.

While the differences between the young and mature immune system continue to be described, understanding the mechanistic basis behind those differences will become paramount. Furthermore, the differences we observe in the young's immune system may be shaped by the simultaneous development of their microbiota. Uncovering how the microbiota impacts its effects will further garner much needed information that will allow for targeted therapeutic approaches not only for the young but for all (Figure 1).

## Author contributions

SS drafted the manuscript. AS revised the manuscript. Both authors contributed to the article and approved the submitted version.

## Funding

AS receives funding from the Burroughs Wellcome Fund, NIH/NIAID K08AI135097, and the Children's Discovery Institute at St. Louis Children's Hospital.

## Conflict of interest

The authors declare that the research was conducted in the absence of any commercial or financial relationships that could be construed as a potential conflict of interest.

## Publisher's note

All claims expressed in this article are solely those of the authors and do not necessarily represent those of their affiliated organizations, or those of the publisher, the editors and the reviewers. Any product that may be evaluated in this article, or claim that may be made by its manufacturer, is not guaranteed or endorsed by the publisher.
